# Prescribing patterns of statins and associated factors among type 2 diabetes mellitus patients attended at Jugol General Hospital in eastern Ethiopia: A cross-sectional study

**DOI:** 10.3389/fcdhc.2023.1061628

**Published:** 2023-03-23

**Authors:** Shambel Nigussie, Fekade Demeke

**Affiliations:** ^1^ Department of Clinical Pharmacy, School of Pharmacy, College of Health and Medical Science, Haramaya University, Harar, Ethiopia; ^2^ Department of Epidemiology, College of Medicine and Health Sciences, Jigjiga University, Jigjiga, Ethiopia

**Keywords:** statin, type 2 diabetes mellitus, cardiovascular disease, low-density lipoprotein, eastern Ethiopia

## Abstract

**Background:**

Most clinical practice guidelines support the use of statins in patients with type 2 diabetes mellitus to lower the risk of cardiovascular disease. However, nothing is known about the prescribing patterns of statins at Jugol General Hospital in eastern Ethiopia.

**Objective:**

This study aimed to assess the prescribing patterns of statins and associated factors among type 2 diabetes mellitus patients attended at Jugol General Hospital in eastern Ethiopia

**Methods:**

A retrospective cross-sectional study was conducted among 423 patients with type 2 diabetes mellitus who received follow-up care from 1 June 2017 to 1 June 2022. The study participants were enrolled consecutively using a convenience sampling technique. The data were extracted from patients’ medical records using a data abstraction checklist. The extracted data were entered into EpiData, version 3.1, and exported to Statistical Package for the Social Sciences (SPSS), version 22, for analysis. Associations were considered to be statistically significant at a *p*-value < 0.05 and presented as adjusted odds ratios and 95% confidence intervals.

**Result:**

The medical records of 423 patients were reviewed. The review revealed that medical records were complete for 410 of these patients, and these records were included in the analysis. The majority of the study participants were female (72.2%) and between the age of 40 and 65 years (61.2%). All of the study participants were eligible for statin prescription; however, statins were prescribed for only 257 (62.7%) study participants. Of the statins prescribed, moderate-dose-intensity statins were prescribed for 40 (15.6%) participants who were at high risk of cardiovascular disease. Atorvastatin was the most commonly (93.3%) prescribed statin. The presence of hypertension, coronary artery disease, and cerebrovascular events was significantly associated with statin prescribing.

**Conclusion:**

The magnitude of prescribing statins for patients with type 2 diabetes mellitus was low in comparison with the clinical practice guidelines recommendation. This finding is alarming and is a call for action to improve the execution of clinical practice guidelines for the benefit of this high-risk population.

## Introduction

Cardiovascular and cerebrovascular disease are more likely to occur in people with type 2 diabetes mellitus (T2DM) than in people without the condition. According to estimates, those who have diabetes and have never had a myocardial infarction have the same risk of experiencing a myocardial infarction as those who do not have diabetes but have had a myocardial infarction in the past (1). The death rate from myocardial infarction is also greater in people with diabetes (2). Cardiovascular disease (CVD) and stroke account for over 60% of deaths in diabetic patients (3). Therefore, it is crucial to manage cardiovascular risk factors, including dyslipidemia. For this reason, optimizing the use of lipid-lowering therapies among T2DM patients should be viewed as one of the key strategies for reducing the total burden of CVD (4).

Numerous clinical trials have demonstrated that the prescription of statin medication improves CVD outcomes in patients with and without coronary heart disease (CHD) (5,6). Each 1 mmol/L (39 mg/dL) drop in low-density lipoprotein (LDL) cholesterol resulted in a 9% reduction in overall mortality and a 13% reduction in vascular mortality, according to a meta-analysis of statin medication (7). Statins are the preferred medication for decreasing LDL cholesterol and for conferring cardioprotection. Two statin dosing intensities are recommended for use in clinical practice: moderate intensity and high intensity. Atorvastatin 10–20 mg, rosuvastatin 5–10 mg, simvastatin 20–40 mg, pravastatin 40–80 mg, lovastatin 40 mg, fluvastatin 40 mg twice daily/80 mg once daily, and pitavastatin 2–4 mg are among the medications used in moderate-intensity statin therapy. Atorvastatin 40–80 mg and rosuvastatin 20–40 mg are used as high-intensity statin therapy. When using a moderate-intensity statin, LDL cholesterol is reduced by 30%–50%, whereas high-intensity statin therapy reduces LDL cholesterol by about 50% (8,9).

According to the 2022 American Diabetes Association standards of medical care in diabetes recommendation, statins are prescribed for the primary or secondary prevention of cardiovascular disease. The criteria for primary prevention, with respect to statin dose intensity, are as follows: patients with diabetes who are 40 years of age or older and are free of atherosclerotic cardiovascular disease receive moderate-intensity statin therapy; and patients who are 40 years of age or older with multiple cardiovascular risk factors (at least two) receive high-intensity statin therapy. The criteria for secondary prevention, with respect to statin dose intensity, are as follows: patients of all ages with diabetes and atherosclerotic cardiovascular disease should receive high-intensity statin therapy. In the case of patients with diabetes and atherosclerotic cardiovascular disease who are considered very high risk using specific criteria, if LDL cholesterol is ≥70 mg/dL on a maximally tolerated statin dose, consider adding additional LDL-lowering therapy such as ezetimibe (10).

The prescribing of statins to patients with T2DM, to lower the risk of cardiovascular disease, is incorporated in most clinical practice guidelines. However, nothing is known about the prescribing pattern of statins at Jugol General Hospital (JGH) in eastern Ethiopia. Hence, this study aimed to assess the prescribing patterns of statins and associated factors among T2DM patients who attended JGH.

## Methods and materials

### Study area, design, and period

A retrospective cross-sectional study was carried out at JGH in Harar, eastern Ethiopia, which is 526 km from Addis Ababa. The hospital provides follow-up care for patients with chronic conditions, including diabetes mellitus. The hospital’s regular diabetic clinic is open every Monday, Tuesday, and Thursday. The clinic offers standard care for patients with diabetes, including blood glucose monitoring, height and weight measurements, medication prescriptions, and dietary recommendations. Data collection was carried out from 18 September to 3 October 2022.

### Source and study population

The source population included all patients with T2DM who had been receiving follow-up care at JGH. All patients with T2DM who had been receiving follow-up care from 1 June 2017 to 1 June 2022 and who fulfilled the inclusion criteria of the study were included in the study population.

### Inclusion and exclusion criteria

Patients with T2DM who had been receiving follow-up care for a minimum of 3 months were included. Women who were pregnant and patients who were under the age of 40 years with no atherosclerotic cardiovascular disease were not included.

### Sample size and sampling procedure

Through the use of a single population proportion formula, the sample size was determined:


$$n=\frac{Z_{\alpha /2}^{2}P(1-P)}{W^{2}},$$

by considering a confidence level of 95% (*Z* = 1.96), a degree of precision of 5%, and *P* = 50% (since there was no previous similar study). The calculated sample size was 384. Allowing 10% contingency gave a final total sample size of 423. Consecutive enrollment of study participants was achieved using the convenience sampling method.

### Data collection procedure

The required information from patients’ medical records was recorded using a data abstraction checklist that was adapted from several pieces of literature (11–15). Sociodemographic characteristics, cardiovascular risk factor, atherosclerotic cardiovascular diseases, chronic diabetes complications, cardiovascular risk stratification, co-medications (i.e., antidiabetic, antihypertensive, and antiplatelet drugs), and laboratory characteristics were all listed on the data abstraction checklist, which was written in English. The sociodemographic characteristics included were sex, age, and place of residence (i.e., in an urban or rural area). Body mass index in the overweight or obese range, hypertension, a history of smoking, and time since T2DM diagnosis of 10 years or more were listed as cardiovascular risk factors. Coronary artery disease, peripheral artery disease, and cerebral vascular events were listed as atherosclerotic cardiovascular diseases. Neuropathy, retinopathy, and nephropathy were included under chronic microvascular complications of diabetes mellitus. Cardiovascular risk was stratified as moderate or high risk (where moderate risk was defined as the presence of one cardiovascular risk factors and high risk was defined as the presence of two or more cardiovascular risk factors). The laboratory record included LDL cholesterol measurements. The data were collected by two pharmacy technicians. To gather the data, patients’ medical record numbers were taken from the diabetic clinic’s registration manual. The chart was retrieved from the medical record room after selecting the patient’s medical record number. Next, using a data abstraction format, the required information was extracted.

### Data quality control

To guarantee the accuracy of the data, a pretest was conducted on 5% of the study participants at Haramaya General Hospital, Haramaya, Ethiopia. To check for consistency in the data abstraction checklist, a pretest was performed on the chart of a randomly chosen patient with T2DM. The data abstraction checklist’s final version was modified to address any errors that were identified during the pretest phase. Prior to the process of collecting data, the data collectors received training. The skilled supervisor made sure that the data were comprehensive and consistent by monitoring and checking them. When managing, storing, and analyzing the data, each piece of information was checked for accuracy and consistency.

### Operational definitions

**Group benefiting from moderate-intensity statin dose**: patients with diabetes aged ≥40 years without atherosclerotic cardiovascular disease.

**Group benefiting from high-intensity statin dose**: all ages of patients with diabetes and atherosclerotic cardiovascular disease or patients aged 40 years and older with multiple (i.e., at least two) cardiovascular risk factors.

### Data analysis and presentations

Statistical Packages for Social Sciences (SPSS) version 22 was used to code, clean, and analyze the acquired data after they had been entered into EpiData statistical program version 3.1. Categorical variables were reported as frequency and percentage, and continuous variables were summarized using means and standard deviations (SDs). To ascertain the relationship between the variables, a binary logistic regression model with bivariate [crude odds ratio (COR)] and multivariate [adjusted odds ratio (AOR)] analysis, along with 95% confidence intervals and *p*-values, was utilized. To compensate for any confounding variables that could influence the prescription of statins, variables with a *p*-value of ≤ 0.25 during a bivariate logistic regression analysis were added to the multivariate logistic regression analysis. The presence and strength of the association between the dependent and independent variables were determined by the AOR and its 95% CI. A *p*-value< 0.05 was taken to mean the presence of statistical significance.

### Ethics considerations

The Institutional Health Research Ethics Review Committee (IHRERC) at the College of Health and Medical Sciences, Haramaya University (reference number: IHRERC/167/2022), provided an ethics clearance letter. Without the patients’ permission, IHRERC permitted the review of patients’ medical records for acceptable reasons (i.e., for research purposes). On behalf of the patients, consent was obtained from the director of JGH. In addition, the director of JGH provided a legal permission letter to collect the data. Information from medical records was kept completely confidential, and the hospital’s head was notified.

## Results

### Sociodemographic and clinical characteristics of the study participants

In this study, the medical records of 423 patients were reviewed. The medical records of 13 of these patients were excluded because they were incomplete. In total, the medical records of 410 patients were used for analysis. Nearly 70% of the study participants (68.1%) were female. The mean (± SD) age of the study participants was 47.19 ± 11.45 years. A total of 251 (61.2%) study participants were aged between 40 and 65 years. The mean duration of diabetes mellitus since diagnosis was 12.3 ± 4.7 years, and two-thirds of participants (274, 66.8%) had been diagnosed with the disease 10 or more years previously.

Of the 410 study participants, around 55% had hypertension and one-third (32.7%) had coronary artery disease. In total, 103 study participants had a documented history of microvascular complications of diabetes mellitus, of whom nearly half (48.5%) had neuropathy (**Table 1**).

**Table 1:** Sociodemographic and clinical characteristics of type 2 diabetes mellitus (T2DM) patients in Jugol General Hospital in Harar in eastern Ethiopia: 2022.

Characteristics	Frequency (%) (*N* = 410)	Statin use, *N* (%)	
		Yes (*n* = 257)	No (*n* = 153)
Sex			
Female Male	298 (72.7) 112 (27.3)	203 (68.1) 54 (48.2)	95 (31.9) 58 (51.8)
Age (years)			
< 40 40–65 ≥ 66	70 (17.1) 251 (61.2) 89 (21.7)	27 (38.6) 158 (62.9) 72 (80.9)	43 (61.4) 93 (37.1) 17 (19.1)
Residence			
Urban Rural	170 (41.5) 240 (58.5)	138 (81.2) 119 (49.6)	32 (18.8) 121 (50.4)
Smoking history			
Yes No	26 (6.3) 384 (93.7%)	12 (46.2) 63 (16.4)	14 (53.8) 321 (83.6)
Body mass index (kg/m^2^)			
Normal Overweight Obese	91 (22.2) 192 (46.8) 127 (31.0)	36 (39.6) 189 (98.4) 32 (25.2)	55 (60.4) 3 (1.6) 95 (74.8)
Duration of diabetes mellitus			
Mean (± SD) < 10 years ≥ 10 years	12.3 ± 4.7 136 (33.2) 274 (66.8)	80 (58.8) 177 (64.6)	56 (41.2) 97 (35.4)
Hypertension			
Yes No	224 (54.6) 186 (45.4)	169 (75.4) 88 (47.3)	55 (24.6) 98 (52.7)
Coronary artery disease			
Yes No	134 (32.7) 276 (67.3)	66 (49.3) 191 (69.2)	68 (50.7) 85 (30.8)
Peripheral artery disease			
Yes No	105 (25.6) 305 (74.4)	92 (87.6) 165 (54.1)	13 (12.4) 140 (45.9)
Cerebrovascular event			
Yes No	85 (20.7) 325 (79.3)	69 (81.2) 188 (57.8)	16 (18.8) 137 (42.2)
Types of microvascular complications			
Neuropathy Nephropathy Retinopathy	*50 (48.5)* *20 (19.4)* *33 (32.0)*	25 (50) 0 0	25 (50) 20 (100) 33 (100)
Low-density lipoprotein level			
Normal High	60 (39.7) 91 (60.3)	35 (58.3) 23 (25.3)	25 (41.7) 68 (74.7)
Cardiovascular risk stratification			
Moderate High	90 (22.0) 320 (78.0)	56 (62.0) 201 (62.8)	34 (37.8) 119 (37.2)

The level of low-density lipoprotein was considered normal if it was ≤ 100 mg/dL. The level of low-density lipoprotein was considered high if it was > 100 mg/dL.

### Prescribed statins and other medications

In this study, 410 (100%) participants were eligible for prescription of statins. However, statins were prescribed to only 257 (62.7%) study participants. The most commonly prescribed type of statin was atorvastatin [240 participants (93.4%)], followed by simvastatin [17 participants (6.6%)]. High-dose statins were prescribed to 161 (62.6%) study participants. More than half of the study participants [i.e., 250 (61.0%)] were on oral hypoglycemic drugs. A total of 139 study participants were taking antiplatelet drugs, of whom almost all (97.8%) were taking aspirin (**Table 2**).

**Table 2:** Prescribing statins and other medications among type 2 diabetes mellitus (T2DM) patients in Jugol General Hospital in Harar, Ethiopia: 2022.

Characteristics	Frequency (%)
Antidiabetic drugs (*n* = 410)	
Oral hypoglycemic Insulin alone Oral hypoglycemic + insulin	250 (61.0) 50 (12.2) 110 (26.8)
Antihypertensive drugs (*n* = 255)	
ACE inhibitors Calcium channel blockers Diuretics	129 (50.5) 62 (24.3) 64 (25.1)
Antiplatelet drugs (*n* = 139)	
Aspirin Clopidogrel	136 (97.8) 3 (2.1)
Statin prescribed (*n* = 410)	
Yes No	257 (62.7) 153 (37.3)
Prescribed type of statin (*n* = 257)	
Atorvastatin Simvastatin	240 (93.4) 17 (6.6)
Statins dose (*n* = 257)	
Moderate High	96 (37.4) 161 (62.6)

ACE, angiotensin-converting enzyme.

**Figure 1:**
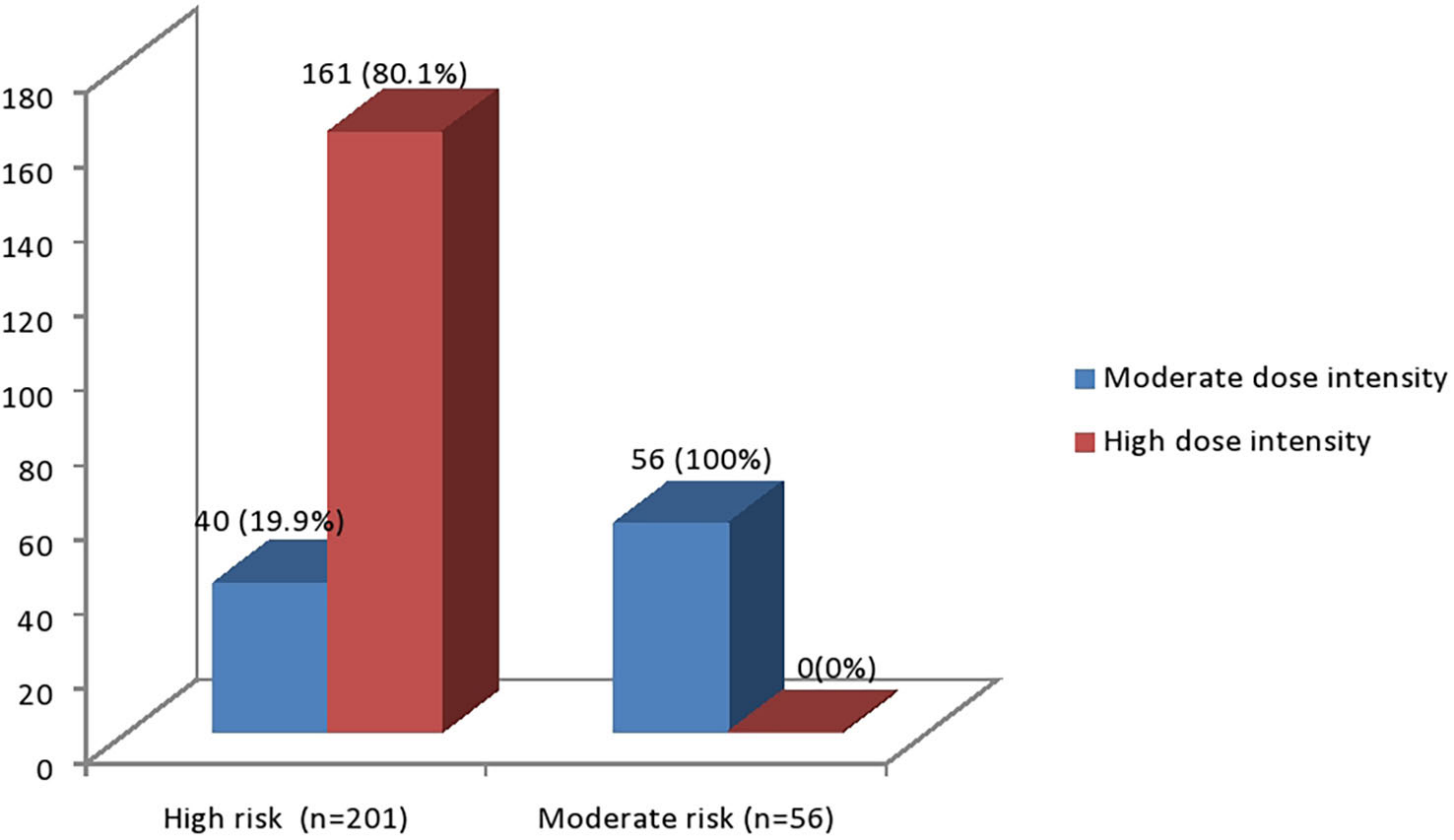
Cardiovascular risk stratification in relation to statin dose-intensity among T2DM patients, 2022.

High-risk individuals were more frequently prescribed high-dose-intensity statins than moderate-dose-intensity statins. However, the opposite was the case for moderate-risk individuals (**Figure 1**).

### Factors associated with prescribing of statins

In this study, comorbid hypertension, coronary artery disease, cerebrovascular events, and age were significantly associated with statin prescription in a multivariate logistic regression analysis. Statins were 6.98 times more likely to be prescribed for participants aged 66 years and older than for participants aged less than 40 years (AOR 6.98, 95% CI 3.51 to 8.78). Similarly, statin prescriptions were 3.61 times more likely for participants who had hypertension than for participants who did not have hypertension (AOR 3.61, 95% CI 2.54 to 5.92). Participants with comorbid coronary artery disease were almost twice as likely to be prescribed statins as their counterparts without coronary artery disease. Participants who had experienced a cerebrovascular event were 3.75 times more likely to have a statin prescription than participants who had not (**Table 3**).

**Table 3:** Factors associated with statin prescription among statin-eligible patients with type 2 diabetes mellitus in Jugol General Hospital in Harar, eastern Ethiopia: 2022.

Variables	Statin prescribed		COR (95% CI)	AOR (95% CI)	*p*-value
	Yes, *n* (%)	No, *n* (%)			
Age (years)					
≥ 66 40–65 < 40	72 (80.9) 158 (62.9) 27 (38.6)	17 (19.1) 93 (37.1) 43 (61.4)	6.74 (3.30 to 8.21) 2.71 (1.57 to 4.67) 1	6.98 (3.51 to 8.78) 2.77 (1.68 to 5.06) 1	0.04* 0.001*
Sex					
Female Male	203 (68.1) 54 (48.2)	95 (31.9) 58 (51.8)	2.29 (0.77 to 3.29) 1	2.36 (0.99 to 3.78) 1	0.12
Residence					
Urban Rural	138 (81.2) 119 (49.6)	32(18.8) 121(50.4)	4.38 (2.76 to 6.94) 1	3.21 (0.97 to 4.56) 1	0.11
Hypertension					
Yes No	169 (75.4) 88 (47.3)	55 (24.6) 98 (52.7)	3.42 (2.25 to 5.20) 1	3.61 (2.54 to 5.92) 1	0.01*
Coronary artery disease					
Yes No	127 (70.6) 130 (56.5)	53 (29.4) 100 (43.5)	1.84 (1.21 to 2.78) 1	1.98 (1.33 to 2.87) 1	0.03*
Cerebrovascular event					
Yes No	69 (81.2) 188 (57.8)	16 (18.8) 137 (42.2)	3.14 (1.74 to 5.65) 1	3.75 (1.89 to 5.93) 1	0.01*

*Statistically significant.

AOR, adjusted odds ratio; COR, crude odds ratio.

## Discussion

This study provides information on prescribing patterns of statins for the prevention of cardiovascular diseases among T2DM patients at Jugol General Hospital.

In this study, 410 (100%) participants were eligible for statin prescriptions. However, statins were prescribed for only 257 (62.7%) study participants. It is encouraging to note that this study figure is greater than those reported from studies carried out in India (55.2%), Germany (25.0%), United Kingdom (33.0%), and Botswana (45.5%) (15–18). One possible explanation for such a discrepancy might be a difference in adherence to recommended guidelines.

In the current study, 37.4% of patients who received statins were prescribed moderate-dose statins, a figure comparable to that reported in a previous study (13). Among those patients who received moderate-dose statins, about 40 (41.7%) were in a high-risk group, contrary to the standard guideline recommendations (10,19). Even though some patients were taking high-intensity statins, the aggregate number of prescriptions was lower than what the guidelines recommend. Evidence suggests that patients who do not receive statin therapy in accordance with recommendations are more likely to experience cardiovascular disease (20). In this study, atorvastatin was shown to be the most commonly prescribed type of statin, which is in line with research results reported from India and Ethiopia (13,16). In contrast, simvastatin was the statin that was most frequently prescribed in a study conducted in Ghana (21). The discrepancy might be due to availability, cost, and physician preference for certain types of statins (22).

Lipid profile results were available for approximately 151 (36.8%) study participants, which is in line with the results of a study conducted in Tanzania (23). This shows a lack of adherence to the accepted recommendations for monitoring lipid profiles, which state that all patients with T2DM should undergo testing a minimum of once a year (19). Hence, to maximize the use of statins in response to cardiovascular disease risk factors, it is important to focus on the routine monitoring of cholesterol levels of patients.

In the current study, statins were more frequently prescribed for patients who had lived with T2DM for 10 or more years since their diagnosis, which is in line with the previous research report (24). In the present study, participants who had a history of hypertension along with diabetes mellitus were prescribed statins significantly more often than those without hypertension. A number of additional studies have also noted this link (25,26). Therefore, having both hypertension and diabetes mellitus should be regarded as an indication that an individual is at high risk for developing cardiovascular diseases, and physicians should act to treat these patients as well as they can before they die from cardiovascular disease (23).

In this study, the presence of coronary artery disease, cerebrovascular events, and hypertension were positively associated with the prescribing of statins. This finding is in line with clinical practice guidelines (10,27). If the guidelines’ recommendations were fully implemented, all this study’s participants would have been eligible for statin prescription owing to the high prevalence of hypertension and other indications among the participants. In line with earlier studies (28,29) in which the odds of being prescribed statins were higher in more elderly individuals, increase in age in this study was associated with statin prescription.

### Limitations of this study

This study was conducted in a single health facility, which makes it difficult to give a conclusion for the general population. The inherent issues with retrospective study designs may potentially be another limitation, as statin prescription may be influenced by other factors not listed in the medical records.

## Conclusion

Overall, the magnitude of prescribing statins for statin-eligible T2DM patients was lower than the target set by clinical practice guidelines. Moderate-dose-intensity statins were prescribed for 40 (15.6%) participants who were at high risk. This finding is alarming and is a call for action to improve the execution of clinical practice guidelines for the benefit of this high-risk population.

## Data availability statement

The original contributions presented in the study are included in the article/supplementary material. Further inquiries can be directed to the corresponding author.

## Ethics statement

The study involving human participants were reviewed and approved by Institutional Health Research Ethics Review Committee (IHRERC). The ethics committee waived the requirement of written informed consent for participation.

## Author contributions

SN and FD conceived the idea and contributed to data review, data analysis, drafting, and revising the final draft. All authors contributed to the article and approved the submitted version.
